# Familial Multiple Coagulation Factor Deficiencies (FMCFDs) in a Large Cohort of Patients—A Single-Center Experience in Genetic Diagnosis

**DOI:** 10.3390/jcm10020347

**Published:** 2021-01-18

**Authors:** Barbara Preisler, Behnaz Pezeshkpoor, Atanas Banchev, Ronald Fischer, Barbara Zieger, Ute Scholz, Heiko Rühl, Bettina Kemkes-Matthes, Ursula Schmitt, Antje Redlich, Sule Unal, Hans-Jürgen Laws, Martin Olivieri, Johannes Oldenburg, Anna Pavlova

**Affiliations:** 1 Institute of Experimental Hematology and Transfusion Medicine, University Clinic Bonn, 53127 Bonn, Germany; Barbara.Preisler@ukbonn.de (B.P.); Behnaz.Pezeshkpoor@ukbonn.de (B.P.); Heiko.Ruehl@ukbonn.de (H.R.); Johannes.Oldenburg@ukbonn.de (J.O.); 2 Department of Paediatric Haematology and Oncology, University Hospital “Tzaritza Giovanna—ISUL”, 1527 Sofia, Bulgaria; bantschev@gmail.com; 3 Hemophilia Care Center, SRH Kurpfalzkrankenhaus Heidelberg, 69123 Heidelberg, Germany; ronald.fischer@srh.de; 4 Department of Pediatrics and Adolescent Medicine, University Medical Center–University of Freiburg, 79106 Freiburg, Germany; barbara.zieger@uniklinik-freiburg.de; 5 Center of Hemostasis, MVZ Labor Leipzig, 04289 Leipzig, Germany; u.scholz@labor-leipzig.de; 6 Hemostasis Center, Justus Liebig University Giessen, 35392 Giessen, Germany; bettina.kemkes-matthes@innere.med.uni-giessen.de; 7 Center of Hemostasis Berlin, 10789 Berlin-Schöneberg, Germany; schmitt@coagumed.de; 8 Pediatric Oncology Department, Otto von Guericke University Children’s Hospital Magdeburg, 39120 Magdeburg, Germany; antje.redlich@med.ovgu.de; 9 Division of Pediatric Hematology Ankara, Hacettepe University, 06100 Ankara, Turkey; suleunal@hacettepe.edu.tr; 10 Department of Pediatric Oncology, Hematology and Clinical Immunology, University of Duesseldorf, 40225 Duesseldorf, Germany; laws@med.uni-duesseldorf.de; 11 Pediatric Hemostasis and Thrombosis Unit, Department of Pediatrics, Pediatric Hemophilia Centre, Dr. von Hauner Children’s Hospital, University Hospital, LMU Munich, 80337 Munich, Germany; martin.olivieri@med.uni-muenchen.de

**Keywords:** blood coagulation disorders, combined deficiency of coagulation factors, genetic testing, NGS, thrombosis

## Abstract

Background: Familial multiple coagulation factor deficiencies (FMCFDs) are a group of inherited hemostatic disorders with the simultaneous reduction of plasma activity of at least two coagulation factors. As consequence, the type and severity of symptoms and the management of bleeding/thrombotic episodes vary among patients. The aim of this study was to identify the underlying genetic defect in patients with FMCFDs. Methods: Activity levels were collected from the largest cohort of laboratory-diagnosed FMCFD patients described so far. Genetic analysis was performed using next-generation sequencing. Results: In total, 52 FMCFDs resulted from coincidental co-inheritance of single-factor deficiencies. All coagulation factors (except factor XII (FXII)) were involved in different combinations. Factor VII (FVII) deficiency showed the highest prevalence. The second group summarized 21 patients with FMCFDs due to a single-gene defect resulting in combined FV/FVIII deficiency or vitamin K–dependent coagulation factor deficiency. In the third group, nine patients with a combined deficiency of FVII and FX caused by the partial deletion of chromosome 13 were identified. The majority of patients exhibited bleeding symptoms while thrombotic events were uncommon. Conclusions: FMCFDs are heritable abnormalities of hemostasis with a very low population frequency rendering them orphan diseases. A combination of comprehensive screening of residual activities and molecular genetic analysis could avoid under- and misdiagnosis.

## 1. Introduction

Familial multiple coagulation factor deficiency (FMCFD) is a rare hemostatic disorder, characterized by the simultaneous reduction of plasma activity of more than one coagulation factor. FMCFDs are caused by genetic defects in one or more underlying genes. Earlier reports of deficiencies in various combinations of coagulation factors led to the classification of FMCFDs into three groups [1,2,3]. In the first group, FMCFDs arise from the coincidental inheritance of single-coagulation-factor deficiencies. This concomitant inheritance could involve two or more single hemorrhagic or thrombotic deficiencies alone or can represent a combination of hemorrhagic and thrombotic disorders [4,5,6]. The second group summarizes cases, where FMCFDs are caused by a single-gene defect. The best known, comprising the vast majority of reported cases of FMCFDs, are combined deficiency of factor V (FV) and factor VIII (FVIII) [7,8] and combined deficiency of vitamin K–dependent clotting factors (VKCFD) [9,10]. The third group includes FMCFDs resulting from cytogenetic abnormalities [11].

Inherited deficiencies of single coagulation factors other than hemophilia A and B and von Willebrand disease are rare disorders, transmitted in an autosomal-recessive pattern. The deficiencies of fibrinogen, prothrombin, FV, factor VII (FVII), factor X (FX), factor XI (FXI), and factor XIII (FXIII) display a variable prevalence ranging from 1:500,000 to 1:2,000,000 [12,13]. The clinical presentation of these disorders varies from asymptomatic, observed for heterozygotes, to severe phenotypes typical of homozygotes or compound heterozygotes [14]. Considering the rare prevalence of single-coagulation-factor deficiencies, combined deficiencies are much rarer except in some ethnic populations due to consanguineous marriages or founder variants [6,15]. Thus, FMCFDs present significant challenges in diagnosis and management and in many cases remain underdiagnosed.

Molecular diagnosis of the single-coagulation-factor deficiencies is based on the identification of pathogenic and likely pathogenic genetic variants in genes encoding corresponding coagulation factors [16]. Causative gene defects can be classified into genetic variants affecting protein biosynthesis/secretion, resulting in low antigen or activity in plasma, or genetic alterations leading to the secretion of a dysfunctional protein. In FMCFDs arising from the coincidental concomitant inheritance of separate coagulation-factor deficiencies, a combination of different disease-causing genetic changes in affected factors can be detected [17,18,19]. In FMCFDs arising from a single-gene defect, such as combined FV and FVIII deficiency and combined deficiency of the vitamin K–dependent factors, genetic variants in the genes encoding proteins responsible for the intracellular trafficking of FV and FVIII, (multiple coagulation factor deficiency 2 (*MCFD2*) and lectin mannose-binding 1 (*LMAN1*)) and genetic variants in genes encoding enzymes involved in post-translational modification and vitamin K metabolism (γ-glutamylcarboxylase (*GGCX*) and vitamin K epoxide reductase complex subunit 1 (*VKORC1*)) are reported [7,20,21,22]. FMCFDs resulting from cytogenetic abnormalities due to the deletion of the end of chromosome 13 cause combined FVII and FX deficiency [18,23]. Similar findings have been reported with case(s) with combined hemophilia A and B [24].

While the diagnosis for a single-coagulation-factor deficiency is often straightforward, diagnosis of FMCFDs can be challenging due to high disease heterogeneity and often indistinct clinical and laboratory features. Frequently, the diagnosis can only be definite when genetic analysis is performed and defects in gene(s) leading to FMCFDs are identified. This paper summarizes genetic and laboratory data of the largest cohort of patients with FMCFDs.

## 2. Experimental Section

### 2.1. Patient Cohort

The study group was composed of 82 unrelated index patients (IPs) from centers in Germany, Switzerland, Turkey, and Lithuania. The inclusion criterion for all participants was the simultaneous reduction of plasma activity of at least two coagulation factors. The mean patients’ age was 26 years, ranged from 3 months to 83 years. Forty-six of them (56%) were male and 36 (44%) were female. Patients’ data were collected by contacting the primary care physicians via questionnaires. Residual coagulation factor activities were analyzed in local labs. The study was approved by the medical ethics committee of the Bonn University of Medical Sciences, and the patients and legal guardians signed a written consent according to the Declaration of Helsinki.

### 2.2. Molecular Genetics Analyses

All genetic analyses were performed in the Department of Molecular Hemostaseology, University Hospital Bonn. Genomic DNA was isolated from peripheral whole blood using the Blood Core Kit (Qiagen, Hilden, Germany). Molecular genetic analyses included the sequencing of all coding regions and intron/exon boundaries of the following genes: *F2*, *F5*, *F7*, *F8*, *F9*, *F10*, *F11*, *F13A1*, *F13B*, *FGA* (fibrinogen alpha chain), *FGB* (fibrinogen beta chain), *FGG* (fibrinogen gamma chain), *VWF*, *GGCX*, *VKORC1*, *LMAN1*, *MCFD2*, *SERPINC1* (serpin family C member 1, antithrombin), *PROS1*, and *PROC*.

The sequencing analyses were carried out on an ABI Prism 3130 genetic analyzer, for Sanger sequencing (Thermo Fisher Scientific, Langenselbold, Germany) and a Mini-Seq genome sequencer (Illumina, Santa Clara, CA, USA) was used for next-generation sequencing (NGS). Data were evaluated by SeqScape Version 2.7 (Thermo Fisher Scientific) and SeqPilot (JSI medical systems, Ettenheim, Germany) software. Primers and conditions are available on request. For the description of sequence variations at the DNA and protein level, the guidelines of the Human Genome Variation Society (HGVS) were used. The genetic variant interpretation and criteria used to establish variant pathogenicity were performed according to the ACMG (American College of Medical Genetics) and AMP (Association for Molecular Pathology) guidelines for the interpretation of sequence variants [25]. The disease causality of genetic variants was compared in the Human Gene Mutation Database (HGMD) [26] and ClinGen database [27].

Large deletions and duplications were analyzed with multiplex ligation-dependent probe amplification (MLPA) analysis or copy number variation (CNV) analysis. MLPA was performed according to the manufacturer’s recommendations, using SALSA MLPA Kits (MRC-Holland, Amsterdam, Netherlands). Amplification products were run on an ABI PRISM 3130XL DNA Sequencer (Thermo Fisher Scientific) with the GeneScan 500 ROX size standard (Thermo Fisher Scientific). Dosage analyses were performed by Coffalyser (V5.2) software (MRC-Holland). CNV evaluation was achieved by SeqPilot (JSI medical systems GmbH).

## 3. Results

### 3.1. Different Mechanisms Causing FMCFDs

We investigated a total of 82 FMCFD index patients with almost exclusively laboratory-diagnosed simultaneous plasma activity reduction of at least two coagulation factors. According to the underlying genetic defects and current classification, patients were divided into three main groups.

#### 3.1.1. Group I: Combined Deficiency Caused by Co-Inherited Deficiency of at Least Two Single Clotting Factors

In this group, we report 52 patients with co-inherited genetic defects in either two pro-coagulant or in two anti-coagulant genes. Patients bearing one genetic alteration in a pro-coagulant gene co-inherited with one genetic defect in an anti-coagulant gene were also included in this group.

In total, 44 patients were identified with a co-inheritance of two pro-coagulant factor deficiencies (Table 1a). Among all patients, the most common detected combined deficiency was that involving FVII. Decreased residual FVII plasma levels (mean 42 IU/dL) and identified *F7* genetic lesions in combination with almost all pro- and anti–coagulant factors were found in 17 patients (33%). Genetic defects in *F7* were detected in the homo-, hetero-, or compound heterozygous state. The clinical symptoms were depicted with different severity. In majority of patients, mild/moderate bleeding events including epistaxis, cutaneous bleeding, oral bleeding, bleeding after surgery or trauma, and menorrhagia were registered. In female patients, menorrhagia was the most common symptom.

In the case of combined FVII and FX deficiency (P5), two genetic alterations were identified in the compound heterozygous state contributing to the low FX residual level and severe clinical presentation. The patient presented severe bleeding episodes (hemarthrosis in the first year of life).

FMCFDs with hemophilia A and B in combination with other coagulation factor disorders represent the second most common combined deficiency in our cohort. In males, the FVIII residual activities, as well as the severity of clinical symptoms were strongly correlated to the *F8* genetic defect. In severe cases, although hemophilia A was combined with other bleeding factor deficiencies, the genetic alterations in the second gene (*FGB* or *FGG* (P29, P30, P31), *F9* (P28)) did not change the severity of the bleeding episodes. Interestingly, in cases where the underlying *F8* genetic variant leads to mild/moderate hemophilia combined with genetic defects in other genes such as *VWF* (P34, P39) and *F11* (P35, P36), bleeding diathesis presented a stronger phenotype compared to the single-gene deficiency. Coincidental inheritance of von Willebrand disease (VWD) with other coagulation factor deficiencies (FV/FVII/FVIII/FIX/FXI/fibrinogen/anti-thrombin) was identified in 10 patients. In a majority of cases, the genetic defects were missense variants in the heterozygous state (except for P49). Seven patients exhibited symptoms of VWD Type 1, two of VWD Type 2 (A and M), and one of VWD Type 3 (Table 1a,b).

In total, six patients showed co-inheritance of a pro- and anti-coagulant factor (Table 1b). Of interest is P49, a VWD Type 3 patient, due to the homozygous large deletion of exon 6. The index patient additionally exhibits an anti-thrombin deficiency, which was proved to be due to a genetic variation in *SERPINC1*. Clinically, this patient was presented with moderate to mild bleeding symptoms (epistaxis, seldom subcutaneous hematomas). A second family member, harboring the same genetic defect in VWF in homozygous state in the absence of anti-thrombin deficiency, suffered from severe bleeding symptoms (severe menorrhagia, hemarthrosis) requiring prophylactic treatment with VWF concentrate. In two patients, a co-inheritance of two anti-coagulant factor deficiencies was identified (Table 1c).

Of interest is also P46 with a co-inheritance of *F7* with *PROC* and *PROS1* defects, where low FVII levels could not attenuate the thrombotic tendency, and the patient was presented with recurrent, severe deep vein thrombosis (DVT). All other coincidentally inherited disorders such as FII/FX, FII/FXI, FV/FX, FIX/fibrinogen, FX/fibrinogen, FX/FXIII, protein C/protein S, and protein S/antithrombin were extremely rare and were detected in one and seldom in more than one patient.

#### 3.1.2. Group II: Combined Deficiency Caused by a Single-Gene Defect

This group involves patients with two monogenic FMCFDs—combined deficiency of FV and FVIII and VKDCF (vitamin K–dependent coagulation factor) deficiency (Table 2). In a single-gene disorder, patients with combined FV and FVIII deficiency are homozygous for loss-of-function pathogenic or likely pathogenic genetic variants in either *LMAN1* or *MCFD2* genes. Eighteen patients exhibited simultaneous reduction of plasma FV and FVIII activities (5% to 49% IU/dL) and mild-to-moderate bleeding symptoms as cutaneous bleeding, epistaxis, bleeding after teeth extraction, surgery, menorrhagia, and increased wound bleeding. Eleven patients (61%) carried homozygous null variants (nonsense, deletion/duplication, and splice-site) in *LMAN1.* In the remaining seven patients, genetic alterations were identified in *MCFD2*. In contrast to *LMAN1*, the genetic variants found in *MCFD2* included null and missense variants.

In our cohort, we identified three patients with combined VKDCF deficiency where the abnormal carboxylase function arises from defects in the gene encoding the γ-gGlutamyl carboxylase (GGCX) enzyme. In all three patients, the disorder arose from genetic variants in compound heterozygous state. The clinical symptoms varied widly from asymptomatic (P72) to intracerebral bleedings (P73).

#### 3.1.3. Group III: Combined Deficiency Arising from Cytogenetic Abnormalities

In this group, we report nine patients who display combined deficiency of FVII and FX due to 13q terminal deletion. The genes encoding these two proteins are mapped to 13q34. All cases were associated with subclinical deficiencies of factors VII and X where the residual plasma activity of each factor was reduced up to 50% of the normal values. The bleeding symptoms were mild, and mainly presented with cutaneous bleedings, epistaxis, oral cavity bleedings, and menorrhagia. All cases show intermediate levels of activity, suggesting heterozygosity (Table 3).

### 3.2. Profile of Genetic Variants in FMCFDs

**Figure 1:**
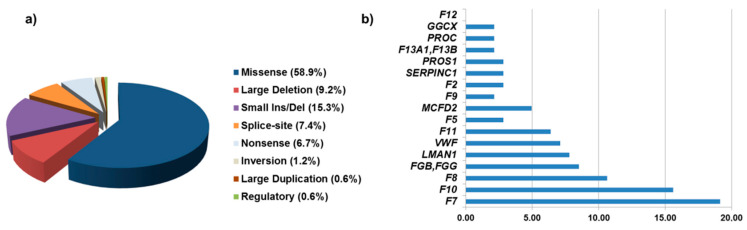
Genetic variants in patients with FMCFD. (**a**) Profile of genetic variants and (**b**) the prevalence of single-factor deficiencies in our study.

Molecular analysis revealed 163 genetic variants in 82 patients with FMCFDs. The majority of genetic defects (58.9%) were missense variants similar to their prevalence in the single-gene deficiency. Interestingly, the proportion of large deletions in comparison to that in the single-gene deficiencies was rather high (9.2%), mostly owing to the cohort of nine patients with combined FVII/FX deficiency due to the distal deletion of chromosome 13. Small deletions/insertions, splice-site, and nonsense variants represent 15.3%, 7.4%, and 6.7% of identified genetic variants in our cohort, respectively (Figure 1a). Splice-site genetic variants were mainly in intronic regions affecting either the donor or acceptor splicing site of the respective gene. In P61, the genetic alteration affected the last nucleotide of the exon 7 of the *LMAN1* gene resulting in no amino acid change but abolishment of the splice-site. Except for the *LMAN1* gene, where only null variants were detected, in all other genes, all types of genetic variants (nonsense, missense, deletion/duplication, and splice-site) were documented. In all, 85 unique genetic variants were already registered in the single-gene database as disease causative, and the remaining 33 (28%) were novel.

In cases with variants with uncertain significance, the causality of the genetic defect was confirmed either by segregation analysis or after exclusion of the identified variant in unaffected family members. In our cohort, 34 different combinations of single-gene deficiency were detected, where factor VII deficiency was frequently involved in combination (Figure 1b). Nearly all coagulation factors (except FXII), with different prevalence, were engaged in this interplay. Moreover, combinations of rather common monogenic disorders (hemophilia A, VWD, factor VII deficiency) were more prevalent than those involving combinations of rare monogenic disorders (factor V, factor II, factor XIII deficiency).

## 4. Discussion

In this study, 82 patients with more than one inherited coagulation factor deficiency were investigated. Majority of our patients constitute the group of inheritance of two different affected genes, followed by FMCFDs due to single-gene defect. A small part represents a group of FMCFDs caused by partial deletion of chromosome 13. The great majority of these deficiencies were associated with bleeding disorders, while the association with thrombotic phenotype was rare.

Bearing in mind, that the rare bleeding disorders (RBDs) represent 3–5% of all inherited deficiencies of coagulation factors, the prevalence of FMCFDs is expected to be much less. Thus, FMCFDs are more sporadic and only isolated case reports are available [23,28,29]. As a consequence of the rarity of these deficiencies, the type and severity of symptoms, the underlying molecular defects, the interaction between the different coagulation factors, and the actual management of bleeding/thrombotic episodes are not well established. The current study presents genetic data on the largest collected and investigated cohort of patients with FMCFD.

Several studies have highlighted the complex biosynthetic pathway of single coagulation factors, which includes several post-translational modifications required for factor secretion and function. Alterations in genes that participate in the protein maturation process, produce and modulate rare coagulation deficiencies as a combined deficiency of coagulation FV and FVIII caused by pathogenic genetic variants in genes encoding proteins involved in the FV and FVIII intracellular transport (*MCFD2* and *LMAN1*) and combined deficiency of all vitamin K–dependent coagulation factors caused by pathogenic genetic variants in the gene coding for GGCX and VKORC1 [8,30,31].

The detected genetic defects in the *LMAN1* gene accounting for the deficiency in all cases were null variants such as nonsense, small deletion/insertion, and splice-site (group II). Clinical symptoms were of a moderate to mild bleeding phenotype. Since its discovery, the combined FV and FVIII deficiency is the most common and well-studied FMCFD. Most described cases come from Mediterranean, Middle Eastern, and South Asian countries likely due to the prevalence of consanguineous marriages in these regions. Sixty percent of our patients had Middle Eastern origin, and all detected variants were in the homozygous state.

The prevalence of large deletions in our cohort was high mainly as a result of FMCFDs arising from cytogenetic abnormalities. This FMCFD was as a consequence of a deletion in the long arm of chromosome 13 involving both FVII- and FX-coding genes, which are known to be in the close vicinity [11,23,32]. Depending on the size of the deletion, other genes, except *F7* and *F10*, could be affected. In such cases, the clinical phenotype could be presented with a large variability including abnormalities such as mental retardation, abnormal fascia or cardiovascular, and musculoskeletal and urogenital developmental anomalies. Such symptoms were not reported in any of our patients, suggesting that the deletion involves the very distal end of chromosome 13, affecting only *F7* and *F10* genes.

Analysis of data of involvement of the single-gene deficiency in FMCFDs showed that nearly all (except FXII) coagulation factors were engaged in this interplay with different prevalences. Moreover, combinations of rather common monogenic disorders (hemophilia A, VWD, factor VII deficiency) were more prevalent than those involving combinations of rare monogenic disorders (factor V, factor II, factor XIII deficiency). In our study, we described 34 different combinations of single-gene deficiency, where factor VII deficiency was frequently involved in combination. An explanation of this finding could be that factor VII deficiency is the most common deficiency (1 in 500,000) among all RBDs [33]. Additionally, FVII deficiency was involved in two groups of FMCFDs—FMCFDs arising from cytogenetic abnormalities and FMCFDs with co-inherited, individual clotting factor genetic defect.

In general, the clinical phenotype in patients with inherited coagulation disorders is related to the residual factor level in the plasma, where the gene variant is the main determinant of such levels. The large heterogeneity of the variant spectrum further provides complexity to the genotype–phenotype relationship. Genetic variants that interrupt protein production (deletion, insertion, splicing variant, nonsense variant) cause a more severe phenotype than non-null and missense variants. However, the relation between genotype and phenotype is not always clearly defined, as the severity of clinical symptoms is highly variable. Even among patients with the same genotype, additional acquired and genetic factors, i.e., functional polymorphisms could modulate the clinical expression of deficiencies. This genotype–phenotype correlation is even more difficult to establish in patients with FMCFDs. Some combinations may increase the severity of bleeding (co-inheritance of two pro-coagulant genes) or of thrombotic events (co-inheritance of two anti-coagulant genes) whereas others may ameliorate the phenotype (co-inheritance of pro-and anti-coagulant genes). As most RBDs are inherited autosomal recessive, the zygosity status of the genetic defect strongly influences the clinical manifestation.

Further complexity in the genotype–phenotype relationship is represented by the delicate balance between coagulation and anticoagulation. Moreover, some coagulation factors, such as thrombin and FV, have both procoagulant and anticoagulant functions [34,35]. It is already documented that co-inheritance of prothrombotic FV Leiden and prothrombin variant (G20210A) mitigate the clinical course in patients with hemophilia [36,37,38]. In combined deficiency of VWF and antithrombin (P49 in our study), both abnormalities counterbalanced each other and brought out a mild hemorrhagic phenotype despite a severe coagulation plasma deficiency caused by the severe genetic defect (homozygous large deletion).

The presence of two clotting factor deficiencies still causes diagnostic and treatment difficulties. The primary diagnosis of most combined deficiencies is usually based on a clinical interpretation of bleeding symptoms as well as performance of standard functional clotting assays.

However, some currently available laboratory assays for coagulation-factor activity are not very sensitive [39]. Moreover, the evaluation of low levels of majority of factors is challenging, leading to false diagnosis or severity assignment [40]. In some cases, the prolonged aPTT can be wrongly interpreted as a monogenic coagulation-factor deficiency and be assumed that the coagulation pattern is due to only one defect. Molecular analysis may clarify some diagnostic uncertainties, but before the era of NGS, the genetic analyses were elaborate. In patients with FMCFD, the availability of NGS has an increasing importance as this technology provides enhanced opportunities simultaneously to detect genetic variants in a large number of genes. Moreover, the NGS technology enables the detection of CNVs, providing a good tool for the detection of large deletions and duplications especially in cases where no MLPA is available. Furthermore, whole-genome or whole-exome sequencing approaches could be applied in an extended research approach to identify new mechanism or new genes involved in the pathogenesis of FMCFDs.

Although NGS facilitates the detection process of genetic, it is often challenging to interpret the causality of all identified genetic variants, especially in case of missense variants. Additionally, the majority of the reported missense variants are unique changes for a given patient, where only experimental studies and investigation of extended family members could contribute immensely to the clarification of the genotype–phenotype correlation. Achieving the correct diagnosis would allow better clinical decision making and disease management, i.e., the choice of treatment, disease prognosis, and family planning.

## 5. Conclusions

FMCFDs are heritable abnormalities of hemostasis with a low overall population frequency, rendering them typically orphan diseases. To avoid misdiagnosis, the combination of comprehensive screening of residual activities, molecular analysis, and family segregation analysis should be performed to achieve the correct diagnosis.

## Figures and Tables

**Table 1:** Laboratory and genetic features of the familial multiple coagulation factor deficiencies (FMCFDs) arising from single, co-inherited clotting factor genetic defect. FMCFDs due to deficiency (**a**) of two pro-coagulant factors, (**b**) two/three pro- and anti-coagulant factors, (**c**) two anti-coagulant factors (m = male, f = female), IU/dL = international units/deciliter, mg/dL = milligram/deciliter. Novel variants are bold.

Patient	Sex	Gene	Nucleotide Position	Aminoacid Exchange	Protein	Laboratory Parameter
**(a) pro-coagulant factor deficiencies**						
P1	m	*F7* *F2*	c.[547G>A];[=] c.[1771G>A];[=]	p.(Asp183Asn) **p.(Gly591Ser)**	FVII Prothrombin	54 IU/dL 38 IU/dL
P2	f	*F7* *F5*	c.[1061C>T];[=] c.[6230T>C];[=]	p.(Ala354Val) **p.(Ile2077Thr)**	FVII FV	58 IU/dL 31 IU/dL
P3	m	*F7* *F5*	c.[215C>G];[=] c.[4900C>T];[=]	**p.(Ser72Cys)** p.(Arg1634Ter)	FVII FV	29 IU/dL 48 IU/dL
P4	m	*F7* *F5*	c.[796G>A];[=] c.[585_586insA];[=]	p.(Ala266Thr) **p.(Gly196Argfs)**	FVII FV	27 IU/dL 42 IU/dL
P5	m	*F7* *F10*	[Large del exons 1-9];[=] c.[1147C>T];[Large del exons 1-6 ]	-- p.(Pro383Ser)	FVII FX	57 IU/dL 2 IU/dL
P6	m	*F7* *F10*	c.[65-3C>T];[Large del exons 4-9] c.[Large del exon 1];[=]	-- --	FVII FX	32 IU/dL 36 IU/dL
P7	f	*F7* *F10*	c.[1061C>T(;)1391delC];[=] c.[706G>A];[=]	p.[(Ala354Val(;)Pro464Hisfs)] p.(Val236 Met)	FVII FX	39 IU/dL 49 IU/dL
P8	f	*F7* *F10*	c.[291+1G>A];[=] c.[301T>C];[=]	-- **p.(Cys101Arg)**	FVII FX	41 IU/dL 48 IU/dL
P9	f	*F7* *F10*	c.[1072A>G];[=] c.[424G>A];[=]	p.(Met358Val) p.(Glu142Lys)	FVII FX	51 IU/dL 47 IU/dL
P10	m	*F7* *F10*	c.[-55C>T];[=] c.[1107G>T];[1108A>T]	-- p.[(**Glu369Asp**(;)Lys370Ter)]	FVII FX	51 IU/dL 25 IU/dL
P11	f	*F7* *F10*	c.[589A>G];[=] c.[424G>A];[=]	p.(Lys197Glu) p.(Glu142Lys)	FVII FX	49 IU/dL 60 IU/dL
P12	f	*F7* *FGA*	c.[1061C>T];[=] c.[103C>T];[=]	p.(Ala354Val) p.(Arg35Cys)	FVII Fibrinogen	35 IU/dL 70 mg/dL
P13	m	*F7* *FGG*	c.[479A>G];[=] c.[(323C>G];[=]	p.(Gln160Arg) p.(Ala108Gly)	FVII Fibrinogen	33 IU/dL 146 mg/dL
P14	f	*F7* *VWF*	c.[817_831del];[=] c.[3518G>T];[=]	p.(Lys273_Asp277del) p.(Cys1173Phe)	FVII VWF (Act./Ag)	66 IU/dL (22/5) IU/dL
P15	f	*F10* *F2*	c.[1333C>G];[=] c.[940C>T];[=]	p.(Arg445Gly) p.(Arg314Cys)	FX Prothrombin	55 IU/dL 65 IU/dL
P16	f	*F10* *F2*	c.[1147C>T];[=] c.[940C>T];[=]	p.(Pro383Ser) p.(Arg314Cys)	FX Prothrombin	42 IU/dL 54 IU/dL
P17	f	*F10* *F5*	c.[1277G>A];[=] c.[5844G>C];[=]	**p.(Arg426His)** ** p.(Trp1948Cys)**	FX FV	50 IU/dL 50 IU/dL
P18	m	*F10* *F13B*	c.[1097G>A];[=] c.[1342C>T];[=]	p.(Arg366His) p.(Pro448Ser)	FX FXIII	45 IU/dL 41 IU/dL
P19	m	*F10* *FGG*	c.[424G>A];[=] c.[323C>G];[=]	p.(Glu142Lys) p.(Ala108Gly)	FX Fibrinogen	64 IU/dL 150 mg/dL
P20	m	*F11* *F2*	c.[901T>C];[=] c.[604C>T];[=]	p.(Phe301Leu) **p.(Gln202Ter)**	FXI Prothrombin	40 IU/dL 51 IU/dL
P21	m	*F11* *F13A1*	c.[1047G>C];[=] c.[233G>A];[233G>A]	**p.(Lys349Asn)** p.(Arg78His)	FXI FXIII	53 IU/dL 3 IU/dL
P22	m	*F11* *FGG*	c.[1724C>T];[=] c.[323C>G];[=]	p.(Ser575Leu) p.(Ala108Gly)	FXI Fibrinogen	42 IU/dL 140 mg/dL
P23	f	*F11* *VWF*	c.[1443delT];[=] c.[3296G>A];[=]	**p.(Ile481Metfs)** p.(Cys1099Tyr)	FXI VWF (Act./Ag)	45 IU/dL (32/25) IU/dL
P24	m	*F11* *VWF*	c.[419G>A];[=] c.[5828G>A];[=]	p.(Cys140Tyr) **p.(Arg1943His)**	FXI VWF (Act./Ag)	51 IU/dL (57/41) IU/dL
P25	f	*FGG* *F5*	c.[323C>G];[=] c.[6528+1_6528+4 delGTAG];[=]	p.(Ala108Gly) --	Fibrinogen FV	160 mg/dL 35 IU/dL
P26	f	*FGB* *VWF*	c.[794C>T];[=] c.[4751A>G];[=]	p.(Pro265Leu) p.(Tyr1584Cys)	Fibrinogen VVWF (Act./Ag)	233 mg/dL (61/38) IU/dL
P27	f	*VWF* *F5*	c.[4094T>C];[=] c.[1669T>C];[=]	p.(Leu1365Pro) **p.(Trp557Arg)**	VWF (Act./Ag) FV	(10/16) IU/dL 41 IU/dL
P28	m	*F8* *F9*	intron 1 inversion c.[835G>A];[0]	-- p.(Ala279Thr)	FVIII FIX	<1 IU/dL 10 IU/dL
P29	m	*F8* *FGG*	c.[7030G>A];[0] c.[323C>G];[=]	p.(Gly2344Ser) p.(Ala108Gly)	FVIII Fibrinogen	<1 IU/dL 124 mg/dL
P30	m	*F8* *FGG*	c.[1978A>C];[0] c.[323C>G];[=]	**p.(Ser660Arg)** p.(Ala108Gly)	FVIII Fibrinogen	<1 IU/dL 101 mg/dL
P31	m	*F8* *FGB*	intron 22 inversion c.[1433G>A];[=]	-- p.(Arg478Lys)	FVIII Fibrinogen	<1 IU/dL 110 mg/dL
P32	m	*F9* *FGB* *FGG*	c.[676C>T];[0] c.[959-7_959-4delCTTTT];[=] c.[323C>G];[=]	p.(Arg226Trp) -- p.(Ala108Gly)	FIX Fibrinogen	<1 IU/dL 120 mg/dL
P33	m	*F8* *F7*	c.[1293G>T];[0] c.[1061C>T];[=]	p.(Leu431Phe) p.(Ala354Val)	FVIII FVII	3 IU/dL 29 IU/dL
P34	m	*F8* *VWF*	c.[5398C>T];[0] c.[6187C>T];[=]	p.(Arg1800Cys) p.(Pro2063Ser)	FVIII VWF (Act./Ag)	4 IU/dL (43/30) IU/dL
P35	m	*F8* *F11*	c.[5793T>A];[0] c.[1075A>G];[=]	**p.(Phe1931Leu)** **p.(Ile359Val)**	FVIII FXI	46 IU/dL 41 IU/dL
P36	m	*F8* * F11*	c.[6325C>T];[0] c.[1540T>C];[=]	p.(Arg2190Cys) **p.(Cys514Arg)**	FVIII FXI	14 IU/dL 30 IU/dL
P37	m	*F8* *F13A1*	c.[752A>C];[0] c.[547A>G];[=]	**p.(His251Pro)** **p.(Ile183Val)**	FVIII FXIII	28 IU/dL 38 IU/dL
P38	m	*F8* *FGG*	c.[121G>T];[0] c.[323C>G];[=]	p.(Gly41Cys) p.(Ala108Gly)	FVIII Fibrinogen	6 IU/dL 170 mg/dL
P39	m	*F8* *VWF*	c.[1481T>C];[0] c.[4636delG];[=]	p.(Ile494Thr) p.(Val1546Ter)	FVIII VWF (Act./Ag)	13 IU/dL (10/15) IU/dL
P40	m	*F8* *F7*	c.[Large dup exon 6];[0] c.[847C>T];[=]	**--** p.(Arg283Trp)	FVIII FVII	26 IU/dL 40 IU/dL
P41	f	*F8* *F7*	c.[1648C>T];[=] c.[509G>A];[=]	p.(Arg550Cys) p.(Arg170His)	FVIII FVII	30 IU/dL 15 IU/dL
P42	f	*F8* *F10*	c.[761A>G];[=] c.[1237G>A];[=]	p.(Asn254Ser) p.(Asp413Asn)	FVIII FX	17 IU/dL 14 IU/dL
P43	f	*F8* *VWF*	c.[1971C>G];[=] c.[4751A>G];[=]	p.(Tyr657Ter) p.(Tyr1584Cys)	FVIII VWF (Act./Ag)	19 IU/dL NA
P44	f	*F9* *VWF*	c.[871G>A];[=] c.[5278G>A];[=]	p.(Glu291Lys) p.(Val1760Ile)	FIX VWF (Act./Ag)	58 IU/dL (40/35) IU/dL
**(b) pro-and anti- coagulant factor deficiencies**						
P45	f	*F9* *SERPINC1*	c.[87A>G];[=] c. [235C>T];[=]	p.(=) p.(Arg79Cys)	FIX Antithormbin	22 IU/dL 49 IU/dL
P46	f	*F7* *PROC* * PROS1*	c.[911C>T];[=] c.[124C>T];[425T>C] c.[1501T>C];[=]	p.(Ala304Val) p.[(Arg42Cys(;)Leu142Pro)] p.(Ser501Pro)	FVII Protein C Free Protein S	51 IU/dL 14 IU/dL 48 IU/dL
P47	m	*FGG* *PROC*	c.[323C>G];[=] c.[1267G>A];[=]	p.(Ala108Gly) p.(Gly423Ser)	Fibrinogen Protein C	130 mg/dL 68 IU/dL
P48	f	*F11* *PROS1*	c.[943G>A];[=] c.[1501T>C];[=]	p.(Glu315Lys) p.(Ser501Pro)	FXI Free Protein S	43 IU/dL 50I U/dL
P49	f	*VWF* *SERPINC1*	[Large del exon 6];[Large del exon 6] c.[133C>T];[=]	-- p.(Arg45Trp)	VWF (Act./Ag) Antithormbin	(1/1) IU/dL 62 IU/dL
P50	f	*F11* *SERPINC1*	c.[1443delT];[=] c.[805G>A];[=]	**p.(Ile481Metfs)** p.(Glu269Lys)	FXI Antithormbin	42 IU/dL 62 IU/dL
**(c) anti-coagulant factor deficiencies**						
P51	f	*PROS1* *PROC*	c.[119G>A];[=] c.[322C>A];[=]	**p.(Arg40His)** p.(His108Asn)	Free Protein S Protein C	57 IU/dL 42 IU/dL
P52	m	*PROS1* *SERPINC1*	c.[1871-2A>G];[=] c.[391C>T];[=]	-- p.(Leu131Phe)	Free Protein S Antithormbin	30 IU/dL 72 IU/dL

**Table 2:** Laboratory and genetic features of the FMCFDs arising from single genetic defects—combined deficiency of FV and FVIII and combined VKDCF deficiency (m = male, f = female); VKDCF—vitamin K–dependent clotting factors, *p*. = synonymous variant, NA = not available due to vitamin k prophylaxis, IU/dL = international units/deciliter.

Patient	Sex	Gene	Nucleotide Position	Aminoacid Exchange	Laboratory Parameter
P53	f	*LMAN1*	c.[275C>G];[275C>G]	**p.(Ser92Ter)**	FV 25 IU/dL FVIII 21 IU/dL
P54	m	*LMAN1*	c.[904A>T];[904A>T]	p.(Lys302Ter)	FV 27 IU/dL FVIII 38 IU/dL
P55	m	*LMAN1*	c.[313G>T];[313G>T]	**p.(Glu105Ter)**	FV 35 IU/dL FVIII 16 IU/dL
P56	m	*LMAN1*	c.[904A>T];[904A>T]	p.(Lys302Ter)	FV 14 IU/dL FVIII 7 IU/dL
P57	m	*LMAN1*	c.[690delT];[690delT]	**p.(Cys230Trpfs)**	FV 8 IU/dL FVIII 11U/dL
P58	m	*LMAN1*	c.[839_840delA];[839_840delA]	p.(Ile281Phefs)	FV 23 IU/dL FVIII 57 IU/dL
P59	m	*LMAN1*	c.[690delT];[690delT]	**p.(Cys230Trpfs)**	FV 9 IU/dL FVIII 12 IU/dL
P60	m	*LMAN1*	c.[912dupA];[912dupA]	p.(Glu305Argfs)	FV 17 IU/dL FVIII 31 IU/dL
P61	m	*LMAN1*	c.[822G>A];[822G>A]	p.(=)	FV 13 IU/dL FVIII 17 IU/dL
P62	m	*LMAN1*	c.[822 +33_822+34insGGTT];[822 +33_822+34insGGTT]	**--**	FV 49 IU/dL FVIII 40 IU/dL
P63	m	*LMAN1*	c.[151_163 insdelTCGAAA];[151_163 insdelTCGAAA]	**p.(Ser51fs)**	FV 31 IU/dL FVIII 16 IU/dL
P64	f	*MCFD2*	c.[407T>C];[407T>C]	p.(Ile136Thr)	FV 5 IU/dL FVIII 21 IU/dL
P65	m	*MCFD2*	c.[266A>C];[266A>C]	p.(Asp89Ala)	FV 16 IU/dL FVIII 8 IU/dL
P66	m	*MCFD2*	c.[249delT];[249delT]	p.(Asp83Glufs)	FV 12 IU/dL FVIII 10 IU/dL
P67	m	*MCFD2*	c.[249delT];[249delT]	p.(Asp83Glufs)	FV 10 IU/dL FVIII 8 IU/dL
P68	m	*MCFD2*	c.[309+1G>A];[309+1G>A]	--	FV 12 IU/dL FVIII 11 IU/dL
P69	f	*MCFD2*	c.[149+1G>A];[149+1G>A]	**--**	FV 7 U/dL FVIII 10 IU/dL
P70	m	*MCFD2*	c.[91_112dup];[91_112dup]	**p.(Met39Glnfs)**	FV 9 IU/dL FVIII 17 IU/dL
P71	f	*GGCX*	c.[944G>A(;)1454G>C]	p.[(Trp315Ter(;)Arg485Pro)]	NA
P72	f	*GGCX*	c.[417T>G(;)1609+3A>G]	**p.(Cys139Trp)**	FII:25 IU/dL, FVII:34 IU/dL FX:18 IU/dL
P73	m	*GGCX*	c.[239C>T(;)1157G>T(;)1595T>C]	p.[(**Pro80Leu**(;)**Gly386Val**(;)Ile532Thr)]	NA

**Table 3:** Laboratory and genetic features of the FMCFDs arising from cytogenetic abnormalities IU/dL = international units/deciliter (m = male, f = female).

Patient	Sex	Gene	Laboratory Parameter
P74	f	*F7* *F10*	58 IU/dL 39 IU/dL
P75	m	*F7* *F10*	42 IU/dL 36 IU/dL
P76	f	*F7* *F10*	35 IU/dL 55 IU/dL
P77	f	*F7* *F10*	42 IU/dL 51 IU/dL
P78	m	*F7* *F10*	25 IU/dL 50 IU/dL
P79	f	*F7* *F10*	22 IU/dL 45IU/dL
P80	f	*F7* *F10*	48 IU/dL 44 IU/dL
P81	f	*F7* *F10*	30 IU/dL 50 IU/dL
P82	f	*F7* * F10*	54 IU/dL 53 IU/dL

## Data Availability

Data is contained within the article.
